# Effect of Timing of Apple Juice Consumption on Postprandial Glycemia: A Randomized Crossover Trial

**DOI:** 10.3390/nu18213657

**Published:** 2026-11-04

**Authors:** Jinru Ren, Yunchu Feng, Ruixin Zhu, Jingjing He, Qi Zhang, Ran Wang, Jie Guo

**Affiliations:** Key Laboratory of Precision Nutrition and Food Quality, Department of Nutrition and Health, China Agricultural University, Beijing 100193, China

**Keywords:** postprandial glycemia, preload, co-ingestion, apple juice, continuous glucose monitoring, second-meal effect

## Abstract

**Background**/**Objectives**: Fruit juice is commonly consumed with meals, yet its liquid matrix and rapidly absorbable carbohydrates may influence postprandial glycemic excursions. Whether altering the timing of fruit juice intake relative to a meal can attenuate postprandial glucose (PPG) remains unclear. This study aimed to investigate the effect of the timing of apple juice consumption on PPG in healthy adults. **Methods**: This randomized, two-period crossover trial (ChiCTR2600121025) enrolled 20 healthy adults in Beijing, China (March 2026), randomized 1:1; 19 with valid continuous glucose monitoring (CGM) data were analyzed. Each consumed 200 mL of 100% apple juice either 30 min before a standardized breakfast (preload) or with the same breakfast (co-ingestion), separated by a 4-day washout. Standardized lunch and dinner were provided. Blinding was not feasible. The primary outcome was breakfast peak glucose measured by CGM; secondary outcomes were incremental area under the curve (iAUC_0–210_), maximum excursion, and the corresponding lunch and dinner outcomes. **Results**: Preloading significantly reduced breakfast peak glucose versus co-ingestion (mean difference 0.51 mmol/L; 95% confidence interval [CI] 0.07–0.94; *p* = 0.025) and breakfast maximum glucose excursion (0.42 mmol/L; 95% CI 0.02–0.82; *p* = 0.040), but not iAUC_0–210_ (0.07 mmol/L·h; 95% CI −0.57 to 0.70; *p* = 0.830); no glycemic outcome differed at lunch or dinner. **Conclusions**: Consuming the same serving of apple juice 30 min before breakfast, rather than with it, reduced breakfast peak glucose and maximum glucose excursion. For individuals who already consume fruit juice, adjusting its timing relative to breakfast reduced breakfast peak glucose and maximum glucose excursion in this small sample; confirmation in larger trials is needed.

## 1. Introduction

The global burden of diabetes continues to rise [1]. About 589 million adults aged 20–79 years were living with diabetes in 2024, and this number is projected to reach 853 million by 2050 [2]. Type 2 diabetes accounts for about 96% of diabetes cases [1]. A metabolic syndrome factor that includes 2 h postload glucose predicts the development of type 2 diabetes [3], and the postload glucose concentration during an oral glucose tolerance test is a strong predictor of future type 2 diabetes [4]. Current diabetes care guidelines recommend managing postprandial glucose (PPG) 1–2 h after meal onset to <180 mg/dL (10.0 mmol/L) [5,6], or a stricter 2 h postprandial glucose threshold of <140 mg/dL (<7.8 mmol/L) [7]. In both patients with type 2 diabetes and individuals without known diabetes, postprandial glucose peaks have been more strongly associated with vascular risk than fasting glucose or HbA1c [8,9]. Flow-mediated dilation (FMD) of the brachial artery is a non-invasive ultrasound measure of endothelium-dependent vasodilation, and reduced FMD reflects endothelial dysfunction, which is considered an early step in the development of atherosclerosis [10,11]. In non-diabetic individuals, postprandial attenuation of FMD was correlated with postprandial glucose fluctuation, insulin resistance and the postprandial serum insulin level, and the greater the glucose fluctuation, the more pronounced the decline in FMD [12]. Consistent with this mechanism, in patients with type 2 diabetes the incremental post-meal glucose peak measured at home was the glycemic variable most strongly correlated with carotid intima-media thickness (IMT) (r = 0.40) [8]. This association persisted across strata of HbA1c, indicating that average glycemia does not adequately capture peak-related risk [8]. This risk signal extends to clinical outcomes. Among 6967 non-critical-care inpatients, each 1 mmol/L higher day-2 peak glucose was associated with a 6% higher adjusted hazard of hospital-acquired complications (HR 1.06, 95% CI 1.04–1.07) [13]. In patients without diabetes, the threshold for increased risk was 13.6 mmol/L [13]. Practical dietary strategies that could attenuate postprandial glycemic excursions may help inform approaches to glycemic management.

Within a meal, consuming protein and vegetables before the carbohydrate component can reduce postprandial glucose and insulin responses [14]. In fruit-preload trials, whole fruits such as apples, oranges, and pears consumed before high-glycemic-index rice meals have been shown to reduce postprandial glycemic excursions [15,16,17]. These effects may be explained by early insulin and incretin responses [18], as well as by delayed gastric emptying, slower intestinal glucose appearance, and the actions of fruit-derived sugars, organic acids, dietary fiber, and polyphenols [19]. Adding 7.5 g of fructose to a 75 g oral glucose load reduced the glucose area under the curve by 19% in healthy adults, possibly through effects on hepatic glucose handling [19]. However, most previous fruit-preload studies have used whole fruit or fruit pieces before rice-based meals [15,16,17,20]. Fruit juice differs from whole fruit in its physical structure, fiber content, and energy density, and it delivers naturally occurring sugars in liquid form [21,22]. Questions remain about whether preload fruit juice could improve PPG. Moreover, prior nutrient intake may improve glycemic responses to later meals, which is also known as the second-meal (subsequent-meal) effect [23,24,25]. Evidence is lacking about whether fruit juice affects glycemic responses to subsequent meals differently when consumed before rather than with a meal.

In this study, we performed a randomized crossover trial among healthy young adults to investigate whether consuming 100% apple juice 30 min before breakfast could improve postprandial glycemic responses compared with consuming the same juice together with breakfast. We also evaluated whether this timing strategy influenced glycemic responses to subsequent meals.

## 2. Methods

This was a single-center randomized crossover trial conducted at China Agricultural University, Beijing, China. Each participant completed two interventions, separated by a 4-day washout period. The trial was conducted between 23 March 2026, and 31 March 2026, in Beijing, China. The study protocol was approved by the China Agricultural University Human Research Ethics Committee (approval code CAUHR-20260211; date of approval: 19 March 2026) and was conducted in accordance with the Declaration of Helsinki. The trial was prospectively registered with the Chinese Clinical Trial Registry on 19 March 2026 [ChiCTR2600121025; https://www.chictr.org.cn/hvshowproject.html?id=297797&v=1.0 (accessed on 20 September 2026)] before enrollment of the first participant. All participants provided written informed consent before enrollment.

### 2.1. Participant Eligibility Criteria

Healthy adults were recruited from China Agricultural University and the surrounding community through study posters and WeChat groups between 9 and 18 March 2026. Eligibility was assessed using a questionnaire, self-reported health history, fasting blood glucose, and routine clinical chemistry tests of liver and kidney function. Inclusion criteria were age 18 to 45 years, no known food allergy, the ability to complete an overnight fast of at least 10 h, and habitual consumption of fruit juice, assessed by questionnaire during screening. Exclusion criteria were pregnancy or lactation; major diseases, including cancer; gastrointestinal disorders, including functional gastrointestinal disorders; heavy physical labor or chronic sleep deprivation; current smoking; diabetes mellitus or any known genetic syndrome or disease affecting glucose tolerance, such as glucose-6-phosphate dehydrogenase deficiency or polycystic ovary syndrome; abnormal liver or kidney function on screening tests; use of medications known to affect glucose tolerance within the previous 30 days, such as thiazide diuretics, glucocorticoids, or spironolactone; and use of medications affecting appetite or gastrointestinal function within the previous 3 months.

### 2.2. Sample Size

Sample size was calculated for the primary outcome, breakfast peak glucose, using a paired *t* test. Based on the 1.2 mmol/L reduction in the incremental postprandial peak reported when 142.8 g of apple (15 g of available carbohydrate) was consumed 30 min before a rice meal rather than with it at an identical carbohydrate content [15], and assuming a preserved difference of 0.7 mmol/L (approximately 60% of that value) and a paired-difference SD of 1.0 mmol/L, 17 completers were required (two-sided α = 0.05, 80% power). Accounting for 15% dropout, 20 participants were enrolled.

### 2.3. Randomization

Participants were assigned in a 1:1 ratio to one of two intervention sequences using a computer-generated randomization sequence. In Sequence A, participants consumed apple juice 30 min before breakfast in Period 1 and consumed apple juice with breakfast in Period 2. In Sequence B, participants received the same interventions in the reverse order. Because the interventions differed in timing, blinding of participants and investigators was not feasible. Statisticians were blinded to participants’ group assignment.

### 2.4. Dietary Interventions

The test beverage was a commercially available 100% apple juice (Longshang Huaniu, Tianshui Great Wall Juice Beverage Co., Ltd., Tianshui, China). Each 200 mL serving provided approximately 430 kJ of energy and 25 g of available carbohydrates.

Participants fasted overnight for at least 10 h before each intervention day. The same standardized meals and beverages were provided on each intervention day; only the timing of apple juice consumption differed between conditions. For the intervention timeline, time 0 was defined as the start of apple juice consumption in the preload condition and as the start of simultaneous apple juice and breakfast consumption in the co-ingestion condition. In the apple-juice preload condition, participants consumed 200 mL apple juice within 5 min at time 0, followed by the standardized solid breakfast 30 min later. In the co-ingestion condition, participants consumed the same serving of apple juice together with breakfast at time 0.

The standardized solid breakfast consisted of 100 g whole-wheat bread and one boiled egg. The standardized lunch consisted of 160 g whole-wheat nut bread, 105 g boiled beef, and 250 g cucumber. The standardized dinner consisted of 100 g whole-wheat bread, 90 g boiled chicken breast, 250 mL milk, and 7 g butter. The composition and nutritional values of the standardized meals are shown in Table 1. Lunch and dinner were served at 210 and 510 min after time 0, respectively. Participants remained on site for the entire intervention day. All meals and the test beverage were provided and consumed under direct observation by the study investigators. Water was permitted ad libitum, but no additional foods or caloric beverages were allowed during the intervention day.

To standardize pre-intervention dietary intake, participants were provided with a standardized evening meal on the day before each intervention visit. This meal consisted of 100 g whole-wheat bread, 90 g boiled chicken breast, and 250 mL milk. During the 24 h before each visit, participants were instructed to avoid alcohol, caffeine-containing beverages, and vigorous physical activity. They were also asked to maintain their habitual sleep schedule and to complete an overnight fast of at least 10 h before each intervention day.

### 2.5. Outcome Measures

The primary outcome was breakfast peak glucose concentration. Secondary outcomes included breakfast postprandial glucose incremental area under the curve (iAUC), breakfast maximum glucose excursion, lunch and dinner iAUC, lunch and dinner peak glucose concentrations, lunch and dinner maximum glucose excursions, intervention-period peak glucose concentration, and intervention-period maximum glucose excursion. Glucose outcomes were derived from continuous glucose monitoring.

Interstitial glucose concentrations were monitored using the FreeStyle Libre Flash Glucose Monitoring System (Abbott Diabetes Care, Alameda, CA, USA). The sensor was applied to the back of the upper arm 3 days before Period 1 and remained in place throughout both intervention periods. Sensors were removed 1 day after completion of Period 2. The system is factory-calibrated and does not require user calibration. Glucose values were recorded by the sensor and exported at 15 min intervals for analysis. Data collected before the first intervention day were used only to confirm sensor wear and were not included in the efficacy analyses.

On intervention days, CGM-derived glucose values were extracted for analysis at predefined time points from 0 to 630 min after time 0. These time points were 0, 15, 30, 45, 60, 90, 120, 150, 180, 210, 225, 240, 255, 270, 300, 330, 360, 390, 420, 450, 480, 510, 525, 540, 555, 570, 600, and 630 min.

Breakfast, lunch, and dinner glucose responses were evaluated over predefined analytical windows of 0–210 min, 210–510 min, and 510–630 min, respectively. The baseline for each window was defined as the glucose value recorded at the start of the corresponding analytical window. The iAUC was calculated using the trapezoidal rule after subtraction of the corresponding pre-prandial baseline value, and incremental values below baseline were set to zero. In formula form, iAUC was calculated as follows:

$$\begin{array}{c} \mathrm{iAUC}=\sum (\frac{G_{i}^{*}+G_{i+1}^{*}}{2}\times \left(t_{i+1}-t_{i}\right))\\ \mathrm{where}:G_{i}^{*}=\max(G_{i}-G_{0},0) \end{array}$$

Peak glucose concentration was defined as the highest glucose value within the corresponding analytical window. Maximum glucose excursion was defined as the difference between the maximum and minimum glucose concentrations observed within the corresponding analytical window. Intervention days with reported intake of non-study foods or caloric beverages, or with unexplained glucose excursions inconsistent with the study meal schedule, were excluded from the efficacy analyses before outcome calculation.

### 2.6. Safety Assessment

Adverse events were assessed by non-systematic (passive) surveillance. At each intervention visit, participants were asked to report any symptoms experienced during the intervention periods, including gastrointestinal discomfort, symptoms of hypoglycemia and local reactions at the CGM sensor site. In addition, a structured gastrointestinal symptom questionnaire was provided after each standardized meal and was completed by participants if any gastrointestinal symptom occurred. The questionnaire covered nausea, bloating, abdominal pain, diarrhea, constipation, and acid regurgitation or heartburn, recorded the severity (mild, moderate or severe) and duration (less than 1 h, 1 to 4 h, or more than 4 h) of any reported symptom, and included an open item for other symptoms.

### 2.7. Statistical Analysis

Continuous variables are presented as mean ± standard deviation for normally distributed data or median with interquartile range for non-normally distributed data. Categorical variables are presented as frequencies and percentages. Continuous variables between sequences were compared using independent-samples *t*-tests or a Wilcoxon rank-sum test. Categorical variables between sequences were compared using Fisher’s exact tests.

Differences between the preload and co-ingestion conditions in iAUC, peak glucose concentration, and maximum glucose excursion were compared using paired *t*-tests for normally distributed data or Wilcoxon signed-rank tests for non-normally distributed data. All statistical analyses were performed using SPSS version 28.0 (IBM Corp., Armonk, NY, USA). A two-sided *p* value < 0.05 was considered statistically significant. All randomized participants who completed both intervention periods were included in the analysis.

The trial used a 2 × 2 crossover design. Because the two sequences received the two conditions in opposite order, the treatment contrast is orthogonal to any period effect by design. No adjustment for the period effect was applied, and this decision was made a priori. The sequence effect was estimated from the between-sequence comparison of within-participant treatment contrasts. For each participant, the treatment contrast was calculated as the peak glucose in the co-ingestion condition minus the peak glucose in the preload condition. The mean contrast of Sequence A was compared with that of Sequence B using an independent-samples *t* test with Welch’s correction. The period effect was estimated from the within-participant difference between periods. For each participant, the difference was calculated as the peak glucose in Period 1 minus the peak glucose in Period 2, so that for Sequence A the difference is the preload value minus the co-ingestion value, and for Sequence B it is the co-ingestion value minus the preload value. The mean of these differences across all participants was tested against zero using a one-sample *t* test. The treatment contribution enters this contrast with opposite signs in the two sequences and therefore largely cancels when they are combined, so the pooled contrast mainly reflects the difference between the first and second periods. Carry-over effects were considered negligible, because the two intervention periods were separated by a 4-day washout and the test beverage was a rapidly absorbed liquid.

The primary analysis used the time origin defined in Section 2.4. In a sensitivity analysis, time 0 was defined as the start of breakfast consumption in both conditions. Breakfast iAUC, peak glucose and maximum glucose excursion were recalculated over the same 210 min window using the same analytical methods as in the primary analysis. The breakfast-aligned sensitivity analysis and the analyses of period and sequence effects were not specified in the registered protocol and are reported as post hoc analyses.

## 3. Results

### 3.1. Participant Flow and Baseline Characteristics

**Figure 1:**
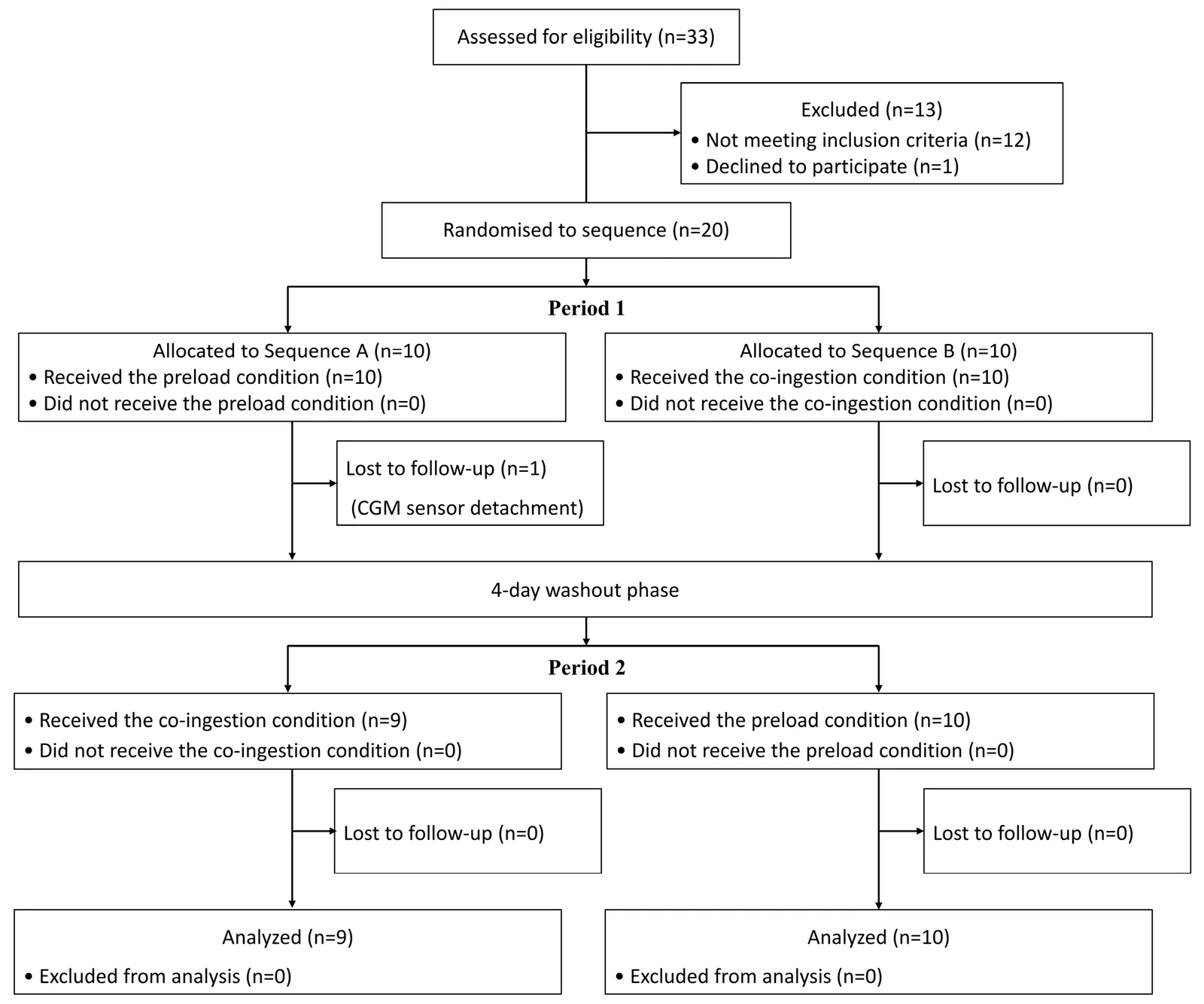
CONSORT flow diagram of the randomized crossover trial.

Of the 33 individuals assessed for eligibility, 13 were excluded, and 20 were randomized (10 per sequence). One participant in Sequence A did not complete Period 1 because the continuous glucose monitoring sensor detached and provided no valid readings, and contributed no data. The remaining 19 participants completed both periods and were included in the analysis (Figure 1).

The median age was 23.0 years (25th, 75th percentile: 23.0, 24.0) in the total sample, 23.0 years (23.0, 24.0) in Sequence A, and 23.5 years (23.0, 24.0) in Sequence B (Table 2). Five participants were male, including 3 of 9 participants in Sequence A and 2 of 10 participants in Sequence B. BMI and baseline glucose were not significantly different between Sequence A and Sequence B.

### 3.2. Postprandial Glycemic Responses

Table 3 shows the postprandial glycemic responses between preload and co-ingestion conditions. Breakfast iAUC_0–210_ did not differ significantly between the co-ingestion and preload conditions (2.59 ± 1.45 vs. 2.52 ± 1.43 mmol/L·h; mean difference: 0.07 mmol/L·h, 95% confidence interval [CI]: −0.57 to 0.70; *p* = 0.830). Breakfast peak glucose concentration was lower in the preload condition than in the co-ingestion condition (6.78 ± 1.02 mmol/L vs. 6.28 ± 0.96; mean difference for co-ingestion minus preload: 0.51 mmol/L, 95% CI: 0.07 to 0.94; *p* = 0.025). Breakfast maximum glucose excursion was also lower in the preload condition than in the co-ingestion condition (2.33 ± 0.97 vs. 2.75 ± 1.20 mmol/L; mean difference for co-ingestion minus preload: 0.42 mmol/L, 95% CI: 0.02 to 0.82; *p* = 0.040; Table 3). Potential period and sequence effects were assessed for the primary outcome. Neither the period effect (−0.19 mmol/L; 95% CI −0.68 to 0.30; *p* = 0.43) nor the sequence effect (−0.43 mmol/L; 95% CI −1.30 to 0.43; *p* = 0.30) was significant (Table S1). In a sensitivity analysis in which the breakfast response was anchored to the start of breakfast consumption in both conditions, breakfast iAUC was lower with preload than with co-ingestion (difference 2.35 mmol/L·h; 95% CI 1.75 to 2.94; *p* < 0.001). The difference in breakfast peak glucose was unchanged from the primary analysis (0.51 mmol/L; 95% CI 0.07 to 0.94; *p* = 0.025), and maximum glucose excursion was likewise lower with preload (difference 0.48 mmol/L; 95% CI 0.07 to 0.90; *p* = 0.024) (Table S2).

During the lunch phase, no significant between-condition differences were observed for iAUC_210–510_ (mean difference: −0.24 mmol/L·h, 95% CI: −1.11 to 0.62; *p* = 0.560), peak glucose concentration (mean difference: −0.09 mmol/L, 95% CI: −0.47 to 0.28; *p* = 0.598), or maximum glucose excursion (mean difference: −0.04 mmol/L, 95% CI: −0.42 to 0.33; *p* = 0.816). During the dinner phase, iAUC_510–630_, peak glucose concentration, and maximum glucose excursion did not differ significantly between conditions. The corresponding mean differences were 0.25 mmol/L·h (95% CI: −0.23 to 0.72; *p* = 0.286), 0.17 mmol/L (95% CI: −0.21 to 0.56; *p* = 0.358), and 0.16 mmol/L (95% CI: −0.18 to 0.51; *p* = 0.335), respectively. For the intervention-period glucose profile, peak glucose concentration was 6.88 ± 0.98 mmol/L in the co-ingestion condition and 6.51 ± 0.94 mmol/L in the preload condition, with a mean difference of 0.37 mmol/L (95% CI: −0.01 to 0.75; *p* = 0.053). Intervention-period maximum glucose excursion was 2.85 ± 1.13 mmol/L and 2.62 ± 0.94 mmol/L, respectively, with a mean difference of 0.24 mmol/L (95% CI: −0.11 to 0.59; *p* = 0.170; Table 3).

### 3.3. Adverse Events

No adverse events or unintended effects were reported by participants during the trial. No participant discontinued the intervention because of an adverse event, and no serious adverse events occurred.

## 4. Discussion

This randomized crossover trial showed that preloading apple juice lowered breakfast peak glucose concentration relative to co-ingestion. Among the secondary outcomes, breakfast maximum glucose excursion was also significantly lower (*p* = 0.040), in the same direction as the primary outcome. No other secondary outcomes differed significantly between conditions. Findings about secondary outcomes are considered as exploratory in this study and further studies with sufficient sample size for these secondary outcomes are warranted. The pattern of results is therefore best understood as a change in the temporal shape of the glucose response rather than a reduction in overall incremental glycemic exposure. This is the expected consequence of the intervention itself: the same amount of juice carbohydrate was consumed in both conditions, and only its timing differed, so part of the carbohydrate load was separated from the solid meal by 30 min. The shift of a portion of the load to an earlier time point attenuates the maximum concentration reached when the remaining carbohydrate is absorbed, while leaving the total incremental exposure over the eating occasion unchanged.

The breakfast-aligned sensitivity analysis illustrates this point and also shows why it cannot serve as the primary comparison. In that analysis, time 0 was redefined as the start of breakfast. The co-ingestion window therefore still contained both the juice and the breakfast, whereas the preload window contained only the standardized breakfast, because most of the juice carbohydrate had already been absorbed before the window began. The two windows therefore differed by approximately 25 g of carbohydrate, and the larger iAUC difference in this comparison (2.35 mmol/L·h; *p* < 0.001) partly reflects this difference in carbohydrate content rather than a pure effect of timing. This analysis should be read as a description of the breakfast response once the preload’s own glycemic excursion has been excluded. The primary analysis remains the appropriate comparison, because it is the only one in which both conditions contain the same carbohydrate load (iAUC difference, 0.07 mmol/L·h; *p* = 0.830). Consuming the juice before the meal introduced the only difference between the conditions, and the absence of any between-condition difference at lunch or dinner indicates that this redistribution did not carry over to subsequent meals.

Previous studies have explored the glycemic benefit of preloading. In the trial with an identical amount of carbohydrate, eating the apple 30 min before the rice significantly reduced postprandial iAUC and peak glucose compared with eating it with the rice [15]. A second set of preload trials extended these observations to three fruits. Among the pear, orange, and apple preloads, the apple preload produced the greatest reduction in postprandial glycemia [16]. Relative to a water preload, the apple preload significantly reduced the postprandial incremental peak glucose and the iAUC [16]. Moreover, different from whole fruit, fruit juice has neither intact cell walls nor appreciable fiber and may have different glycemic benefits of preloading. Intact plant cell walls limit amylase access to intracellular sugars and slow their release [26]. Dietary fiber has been reported to delay gastric emptying and to reduce postprandial glycemia [27]. However, evidence about the effect of preloading fruit juice on PPG is lacking. In this study, we found that preloading reduced breakfast peak glucose by 0.51 mmol/L and maximum glucose excursion by 0.42 mmol/L, albeit not for breakfast iAUC_0–210_. One study of non-critical-care inpatients reported that each 1 mmol/L higher day-2 peak blood glucose was associated with a 6% higher adjusted hazard of hospital-acquired complications (HR 1.06, 95% CI 1.04–1.07) [13]. No study has established whether a 0.5 mmol/L reduction in peak glucose is clinically meaningful, and larger randomized trials are needed to address this question. Our findings indicate that, for individuals who already drink juice with breakfast, moving the same serving to about 30 min before the meal lowered breakfast peak glucose and maximum glucose excursion without changing total incremental glycemic exposure.

A further question is whether preloading juice could influence glycemia beyond the immediate meal. The second-meal effect refers to the phenomenon whereby one meal can influence the blood glucose response to carbohydrate at a subsequent meal [28,29]. This benefit may even extend to a third meal when fermentable substrate persists in the colon [29]. The proposed mechanism is colonic fermentation of indigestible carbohydrate into short-chain fatty acids, which suppress circulating non-esterified fatty acids and slow gastric emptying [30]. Whether a fruit juice preload has a second- or third-meal effect had not been explored. A second-meal effect is unlikely in this setting for three reasons. Apple juice contains negligible fiber and resistant starch and therefore provides little fermentable substrate [30]; its 25 g of free sugars are absorbed early, and the standardized lunch and dinner supplied much larger carbohydrate loads (74.4 g and 61.3 g). The effect of apple juice preloading therefore appears to be confined to the breakfast meal.

This study has several limitations. First, the sample size of this study was small (*n* = 19 analyzed), and the trial was designed to detect a difference of 0.7 mmol/L in peak glucose with 80% power. The observed mean difference was 0.51 mmol/L (95% CI 0.07 to 0.94), which is compatible with effects ranging from negligible to larger than the difference the trial was designed to detect. The clinical significance of this finding should therefore be interpreted cautiously and confirmed in larger trials. Second, participants were young, metabolically healthy adults, and individuals with diabetes or other conditions affecting glucose tolerance were excluded. The findings may therefore not be generalizable to other populations, such as older adults or adults with impaired glucose metabolism, and further studies with varied populations are warranted. Third, double blinding was not feasible because participants necessarily knew whether apple juice was consumed before or with breakfast. However, glucose outcomes were collected objectively by CGM, and the statistician analyzed coded groups without knowledge of allocation. Finally, glucose was measured by CGM in interstitial fluid rather than in plasma, and values were analyzed at 15 min intervals, so the true maximum may have occurred between the analyzed time points. These characteristics were identical in both conditions and would tend to attenuate rather than create a between-condition difference.

## 5. Conclusions

In conclusion, compared with co-ingestion, preloading with 100% apple juice modified the shape of the breakfast glucose response in healthy young adults, reducing the peak and maximum glucose excursion without changing total incremental exposure. These findings come from a small sample size trial in young, healthy adults and should be confirmed in larger studies with varied populations, before clinical recommendations are made.

## Figures and Tables

**Table 1:** Composition and nutritional values of standardized meals.

Meal	Food Item	Amount (g or mL)	Energy (kJ)	Protein (g)	Fat (g)	Carbohydrate (g)
Breakfast	Whole-wheat bread	100 g	1092.0	11.50	0.00	49.30
	Boiled egg	35 g	200.2	4.76	2.90	0.70
	Apple juice	200 mL	430.0	0.00	0.00	25.00
	Total		1722.2	16.26	2.90	75.00
Lunch	Whole-wheat nut bread	160 g	2668.8	19.20	29.12	74.40
	Boiled beef	105 g	632.1	28.98	3.78	0.00
	Cucumber	250 g	131.1	2.07	0.46	5.75
	Total		3432.0	50.25	33.36	80.15
Dinner	Whole-wheat bread	100 g	1092.0	11.50	0.00	49.30
	Chicken breast	90 g	561.6	29.52	1.62	0.00
	Milk	250 mL	710.0	8.00	10.00	12.00
	Butter	7 g	215.7	0.00	5.81	0.04
	Total		2579.3	49.02	17.43	61.34

Nutritional composition: Calculated using the China Food Composition Tables.

**Table 2:** Baseline characteristics of participants by randomized sequence.

Variable	Total (*n* = 19)	Sequence A (*n* = 9)	Sequence B (*n* = 10)	*p* Value ^1^
Age, median (25th,75th), years	23.0 (23.0, 24.0)	23.0 (23.0, 24.0)	23.5 (23.0, 24.0)	0.467
Male, *n* (%)	5 (26.3)	3 (33.3)	2 (20.0)	0.628
BMI, mean ± SD, kg/m^2^	22.1 ± 4.0	20.9 ± 2.5	23.1 ± 4.9	0.234
Baseline glucose, mean ± SD, mmol/L	5.12 ± 0.57	5.07 ± 0.51	5.17 ± 0.64	0.703

Abbreviations: BMI, body mass index; SD, standard deviation. ^1^ *p* values were calculated using the Wilcoxon rank-sum test for age, independent-samples *t*-tests for BMI and baseline glucose, and Fisher’s exact test for sex.

**Table 3:** Postprandial and daily glycemic responses by intervention condition.

Phase	Metric	Co-Ingestion, Mean ± SD	Preload, Mean ± SD	Difference (95% CI) ^1^	*p* Value ^2^
Breakfast	iAUC_0–210_ (mmol/L·h)	2.59 ± 1.45	2.52 ± 1.43	0.07 (−0.57, 0.70)	0.830
	Peak (mmol/L)	6.78 ± 1.02	6.28 ± 0.96	0.51 (0.07, 0.94)	0.025
	maximum glucose excursion (mmol/L)	2.75 ± 1.20	2.33 ± 0.97	0.42 (0.02, 0.82)	0.040
Lunch	iAUC_210–510_ (mmol/L·h)	3.35 ± 1.78	3.60 ± 1.72	−0.24 (−1.11, 0.62)	0.560
	Peak (mmol/L)	6.05 ± 0.93	6.15 ± 0.89	−0.09 (−0.47, 0.28)	0.598
	maximum glucose excursion (mmol/L)	1.96 ± 0.94	2.01 ± 0.92	−0.04 (−0.42, 0.33)	0.816
Dinner	iAUC_510–630_ (mmol/L·h)	1.41 ± 0.90	1.16 ± 1.03	0.25 (−0.23, 0.72)	0.286
	Peak (mmol/L)	5.93 ± 0.82	5.76 ± 0.82	0.17 (−0.21, 0.56)	0.358
	maximum glucose excursion (mmol/L)	1.39 ± 0.69	1.23 ± 0.64	0.16 (−0.18, 0.51)	0.335
Intervention-period glucose profile ^3^	Peak (mmol/L)	6.88 ± 0.98	6.51 ± 0.94	0.37 (−0.01, 0.75)	0.053
	maximum glucose excursion (mmol/L)	2.85 ± 1.13	2.62 ± 0.94	0.24 (−0.11, 0.59)	0.170

Abbreviations: CI, confidence interval; iAUC, incremental area under the curve; and SD, standard deviation. ^1^ Differences are expressed as co-ingestion minus preload; positive values indicate higher values in the co-ingestion condition. ^2^ *p* values were calculated using paired *t*-tests comparing the preload and co-ingestion conditions. ^3^ Intervention-period glucose profile metrics were calculated using glucose values recorded from 0 to 630 min after the condition-specific time 0.

## Data Availability

De-identified participant data, trial protocol, and statistical analysis plan will be made available to qualified researchers upon request to the corresponding author (Jie Guo, guojie@cau.edu.cn), subject to approval by the trial steering committee and a signed data access agreement.

## Supplementary Materials

The following supporting information can be downloaded at: https://www.mdpi.com/article/10.3390/nu18213657/s1, Table S1: Assessment of sequence and period effects on the primary outcome (breakfast peak glucose); Table S2: Sensitivity analysis of breakfast glycemic responses with the analytical window anchored to the start of breakfast consumption in both conditions; Figure S1: Whole-day glycemic responses aligned to the start of intake (primary analysis); Figure S2: Whole-day glycemic responses aligned to the start of breakfast (post hoc sensitivity analysis).

## Abbreviations

The following abbreviations are used in this manuscript:

PPG	Postprandial glucose
CGM	Continuous glucose monitoring
CI	Confidence interval
sauce	Incremental area under the curve
SD	Standard deviation
BMI	Body mass index
