# Supplementary material for: Effect of Timing of Apple Juice Consumption on Postprandial Glycemia: A Randomized Crossover Trial
Source: Nutrients. 2026 Nov 4;18(21):3657. doi: 10.3390/nu18213657 (PMC13765375; doi:10.3390/nu18213657)
Supplement: Supplementary file 1 [file nutrients-18-03657-s001.zip › nutrients-4610716-supplementary.pdf]

Table S1. Assessment of sequence and period effects on the primary outcome (breakfast peak glucose).

Comparison	Mean ± SD [a]	Difference (95% CI)	*P* value [b]
Sequence A vs Sequence B (mmol/L)	0.73 ± 0.85 vs 0.30 ± 0.93	−0.43 (−1.30 to 0.43)[c]	0.30
Period 1 vs Period 2 (mmol/L)	−0.19 ± 1.02	−0.19 (−0.68 to 0.30)[d]	0.43

[a] For the sequence comparison, the treatment contrast was calculated for each participant as the co-ingestion peak glucose minus the preload peak glucose (mmol/L); values are mean ± SD within each sequence (n = 9 and 10). For the period comparison, the period difference was calculated for each participant as the peak glucose in Period 1 minus the peak glucose in Period 2 (mmol/L), irrespective of the condition received; values are mean ± SD across all participants (n = 19).

[b] Sequence effect: independent-samples t test with Welch's correction. Period effect: one-sample t test of the pooled mean against zero.

[c] Difference between sequences (Sequence B - Sequence A).

[d] Mean difference from zero (95% CI).

Table S2. Sensitivity analysis of breakfast glycemic responses with the analytical window anchored to the start of breakfast consumption in both conditions.

Metric	Co-ingestion, mean ± SD	Preload, mean ± SD	Difference (95% CI) [a]	*P* value [b]
Breakfast $iAUC_{0-210}$ (mmol/L·h)	2.59 ± 1.45	0.24 ± 0.50	2.35 (1.75, 2.94)	<0.001
Breakfast peak (mmol/L)	6.78 ± 1.02	6.28 ± 0.96	0.51 (0.07, 0.94)	0.025
Breakfast maximum glucose excursion (mmol/L)	2.75 ± 1.20	2.27 ± 0.96	0.48 (0.07, 0.9)	0.024

[a] Co-ingestion minus preload; positive values indicate higher values in the co-ingestion condition.

[b] Paired t-tests or Wilcoxon signed-rank tests as appropriate. No adjustment was made for multiple comparisons; the analysis is exploratory.

Figure S1. Whole-day glycemic responses aligned to the start of intake (primary analysis).

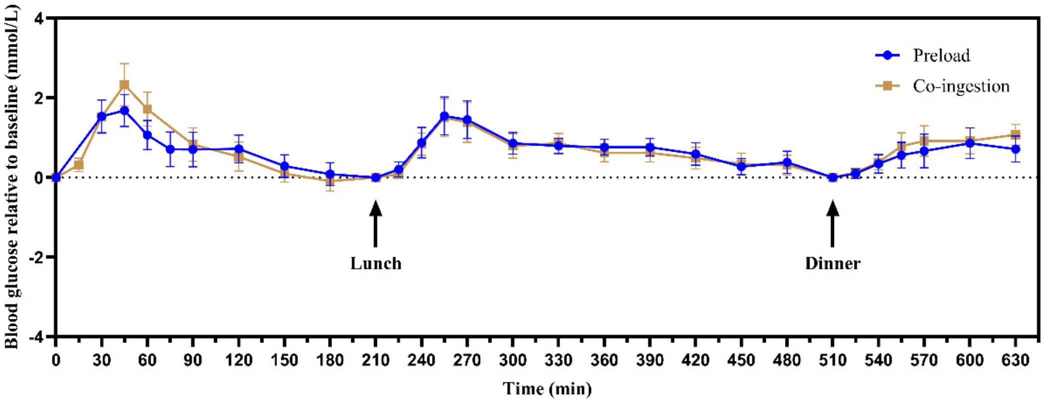

Note: Mean changes in blood glucose concentration over 630 min in the preload (blue circles) and co-ingestion (beige squares) conditions (n = 19), with 95% confidence intervals shown as vertical bars. Time 0 is the start of intake in each condition. In the preload condition, time 0 is the start of apple juice consumption, and the standardized breakfast was consumed 30 min later; in the co-ingestion condition,

time 0 is the start of simultaneous consumption of the apple juice and the standardized breakfast, so the breakfast was consumed at time 0. Values are expressed as changes from the concentration at the start of each meal phase, that is, at 0 min for breakfast, 210 min for lunch and 510 min for dinner. Arrows indicate lunch (210 min) and dinner (510 min). Blood glucose was measured by interstitial continuous glucose monitoring (FreeStyle Libre) at 15-min intervals. Data are means with 95% confidence intervals.

Figure S2. Whole-day glycemic responses aligned to the start of breakfast (post hoc sensitivity analysis).

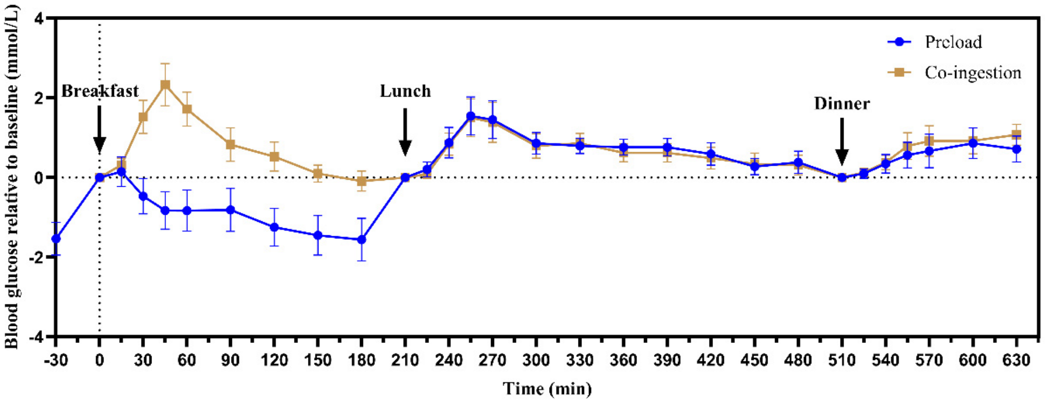

Note: Mean changes in blood glucose concentration over 630 min in the preload (blue circles) and co-ingestion (beige squares) conditions (n = 19), with 95% confidence intervals shown as vertical bars. Time 0 (vertical dotted line) is the start of breakfast consumption in both conditions. In the preload condition, 200 mL of 100% apple juice was consumed at −30 min, that is, 30 min before time 0, so the rise from −30 to 0 min reflects the absorption of the juice; the segment from −30 to 0 min is shown for reference only and is not included in the breakfast-aligned calculation. In the co-ingestion condition, the juice was consumed with the breakfast at time 0. Values are expressed as changes from the concentration at the start of each meal phase, that is, at 0 min for breakfast, 210 min for lunch and 510 min for dinner. Arrows indicate breakfast (0 min), lunch (210 min) and dinner (510 min). Blood glucose was measured by interstitial continuous glucose monitoring (FreeStyle Libre) at 15-min intervals. Data are means with 95% confidence intervals.
